# Problem-solving in groups of common marmosets (*Callithrix jacchus*): more than the sum of its parts

**DOI:** 10.1093/pnasnexus/pgac168

**Published:** 2022-09-14

**Authors:** Sandro Sehner, Erik P Willems, Lucio Vinicus, Andrea B Migliano, Carel P van Schaik, Judith M Burkart

**Affiliations:** Department of Anthropology, University of Zurich, Winterthurerstrasse 190, CH-8057 Zurich, Switzerland; Department of Anthropology, University of Zurich, Winterthurerstrasse 190, CH-8057 Zurich, Switzerland; Department of Anthropology, University of Zurich, Winterthurerstrasse 190, CH-8057 Zurich, Switzerland; Department of Anthropology, University of Zurich, Winterthurerstrasse 190, CH-8057 Zurich, Switzerland; Department of Anthropology, University of Zurich, Winterthurerstrasse 190, CH-8057 Zurich, Switzerland; Center for the Interdisciplinary Study of Language Evolution (ISLE), University of Zurich, Affolternstrasse 56, CH-8050 Zurich, Switzerland; Department of Anthropology, University of Zurich, Winterthurerstrasse 190, CH-8057 Zurich, Switzerland; Center for the Interdisciplinary Study of Language Evolution (ISLE), University of Zurich, Affolternstrasse 56, CH-8050 Zurich, Switzerland

**Keywords:** group problem-solving, nonhuman primates, cooperative breeding, social learning, socially induced perseverance

## Abstract

Human hypercooperativity and the emergence of division of labor enables us to solve problems not only effectively within a group but also collectively. Collective problem-solving occurs when groups perform better than the additive performance of separate individuals. Currently, it is unknown whether this is unique to humans. To investigate the evolutionary origin of collective problem-solving and potential precursors, we propose a continuum of group effects on problem-solving, from simple to complex ones, eventually culminating in collective problem-solving. We tested captive common marmosets with a series of problem-solving tasks, either alone or in a group. To test whether the performance of a group was more than the sum of its parts, we compared real groups to virtual groups (pooled scores of animals tested alone). Marmosets in real groups were both more likely to solve problems than marmosets within the virtual groups and to do so faster. Although individuals within real groups approached the problem faster, a reduction in neophobia was not sufficient to explain the greater success. Success within real groups arose because animals showed higher perseverance, especially after a fellow group member had found the solution in complex tasks. These results are consistent with the idea that group problem-solving evolved alongside a continuum, with performance improving beyond baseline as societies move from social tolerance to opportunities for diffusion of information to active exchange of information. We suggest that increasing interdependence and the adoption of cooperative breeding pushed our ancestors up this scale.

Significance Statement In humans, solving a problem in a group can facilitate performance beyond simple additive effects. Currently, we do not know if groups of nonhuman animals can also solve problems more efficiently than a comparable number of solitary individuals. We demonstrate that in a variety of novel problems, real groups of common marmosets (*Callithrix jacchus*) can, like humans, outperform the mere sum of the individual performances pooled together as virtual groups. We also demonstrate that this effect is not driven by neophobia but rather by an increase in perseverance of individuals within groups. Our results indicate that passive diffusion of information mediated by high levels of social tolerance is sufficient for individuals in a group to gain an advantage over solitary animals.

## Introduction

Humans can process information and solve problems collectively, which greatly facilitates cumulative cultural evolution (1) and has led to a unique way of task specification and systematic division of labor among group members (1, 2). Our species thus evolved what can be defined as “true collective problem-solving,” because the division of labor has become so pronounced that single individuals may no longer be able to understand each step toward the final solution. But even small groups often solve complex problems faster and more precisely than the best individuals working on their own (3–5), leading some to argue that collaborating human groups achieve a form of “collective intelligence” (6–11).

It is difficult to gauge how “true collective problem-solving” evolved because existing studies are strongly biased toward humans (3–14), and based on an arbitrary dichotomy in “true collective problem-solving” being present or not. Here we argue that a perspective on group problem-solving as a continuum with “true collective problem-solving” at its tip (Table 1) will be helpful to investigate its evolutionary origin by studying nonhuman animals. In particular, this approach will allow us to investigate if precursors to “true collective problem-solving” are present among nonhuman animals (henceforth “animals”). This is not unlikely, given that crucial elements such as innovation and social learning (SL) are common across animals (15–23), as, for instance, evident in the widespread occurrence of cultural variation in various animal species (24–26).

**Table 1.** The collective problem-solving continuum.

Mechanism	Definition	Measure
True collective problem-solving	Individuals perform distinct but complementary actions, all necessary to achieve a common goal (“division of labor”).	Individuals in real groups collaborate by **combining their skills/knowledge** to solve a given problem. Individuals in virtual groups are unlikely to solve the problem. Note that for conjunctive tasks each individual in a virtual group should be capable of solving a subtask that is required to solve the problem as a whole.
Information donation	An experienced individual acts in a direct way specifically toward a naïve individual with the intent to communicate information.	A knowledgeable demonstrator within real groups **shows a behavioral change conducive to information donation** only in the presence of naïve individuals but not in their absence. The behavioral change comes with some cost (e.g. longer solving time) to the demonstrator.
Imitation	Copying the exact actions of a novel behavior.	Individuals within real groups **match the exact behavior** of a demonstrator (e.g. in a two-way task). Individuals within virtual groups may solve the problem but not match the exact behavior of others.
Socially induced perseverance	Observing a demonstrator solving a novel problem increases the likelihood that the observer will increase its goal directed behavior as well.	Individuals within real groups increase their **goal directed exploration** subsequently to the success of a conspecific.
Stimulus enhancement	Observing a demonstrator interacting with a stimulus increases the likelihood that the observer will interact with the stimulus as well.	Individuals within real groups are more likely to **explore a stimulus longer** than individuals within virtual groups.
Social facilitation	Mere presence of conspecifics increases the level of arousal of an individual.	Individuals within real groups show a **reduced neophobia** compared to individuals within virtual groups.
Pool of competences; Skill pool	Larger groups of animals are more likely to contain a knowledgeable individual. Increasing group size leads to increasing competition, which forces individuals to specialize on distinct foraging techniques.	Individuals within virtual groups are **as fast and as likely to solve** a novel problem than individuals within a real group.
Competition; Scrounging; Tactical deception	Animals show antagonistic behavior and dominants monopolize resources. Animals within a social environment are less likely to approach a novel problem and “negotiate” risk taking. Animals use honest cues to mislead conspecifics and conceal own knowledge about the environment.	Individuals in virtual groups **are faster and more likely to solve** a novel problem than individuals in real groups (provided novelty avoidance plays no role).

At the bottom are mechanisms that negatively affect problem-solving in the group, at the top mechanisms that positively affect it, with increasing magnitude. Based on the level of intragroup competition and the underlying mechanism of SL individuals in a social environment will fit along this continuum, measurable by comparing animals in real and virtual groups. Lower levels of competition and more sophisticated mechanisms of SL allow groups to solve more complex problems. Except true collective problem-solving, each mechanism can be tested with either a disjunctive, additive or compensatory task design 27). True collective problem-solving should be tested with a conjunctive task design, where each individual needs to solve a subproblem to complete the project.

Previous studies with animals have either focused on collective decision making (27, 28), especially in social insects (29, 30), or provided dyads or groups with problems (for reviews see 19, 20). Relatively few studies have specifically examined problem-solving in a group (31–35). Those that have typically focused on whether larger groups are better than smaller groups. However, these studies did not investigate whether larger groups were indeed more likely to solve novel problems compared to the same number of individuals independently solving the problem. Group problem-solving studies in animals have often assessed the pool of competence or skill pool hypotheses, which predict that since larger groups contain a higher diversity of skills, tendencies or experience, the probability of solving a problem increases in a nonlinear fashion with group size (32–33). By comparing groups of varying sizes (31–33, 36–38), it has been shown that problem-solving success can indeed increase with group size (31–33), even though this does not always happen (36–38).

Group size comparisons thus say less about the collective propensity to innovate than about the effect of a larger number of individuals being sampled. A recent study has indicated that the probability of solving a problem will increase nonlinearly for easy and complex problems with increasing group size (39). The probability of success per group was measured as innovation rate powered by the group size times the number of individual attempts. Thus, this relationship holds without invoking a pool of competence effect (39). Other simulations came to the same conclusion that comparisons at a group level (including group size comparisons) can help to distinguish group size—associated costs from group size—associated benefits, without pinning down the exact mechanism (40). Hence, group comparisons can help to identify group size dependent costs, like competition, but fail to pinpoint why groups are better problem solvers when compared to solitary individuals, and thus fail to explain the advantage of the collective.

A complementary approach to test the effect of the group on problem-solving competence is to compare the individual performance of animals in a social environment (i.e. in a group) and in an asocial environment (i.e. alone). To estimate the influence of the group beyond the sampling bias described by the pool of competence hypotheses (i.e. that sampling more individuals in larger groups increases the likelihood to find a particularly skilled, motivated or experienced individual), we compared the performance of individuals in the social environment condition (henceforth “real groups”) with the individual performance in the asocial environment condition after pooling animals tested alone into virtual groups of the same size (see the “Materials and methods” section).

Table 1 summarizes different effects that the presence of a group can have on problem-solving. The bottom row describes mechanisms that can lead to negative effects, followed in ascending order with mechanisms leading to positive effects. Since these effects can eventually play out simultaneously, they will lead to a net observable effect of being in a group on an individual's problem-solving ability. Among negative effects, intragroup competition can reduce an individual's ability to focus on the problem or even result in agonistic behaviors. Likewise, the risk of scrounging can reduce the effort an individual puts into solving a given task (37) or even motivate individuals to conceal their knowledge about a hidden resource (41, 42), thereby decreasing the opportunity for SL in others. As a result, an individual may do worse in a group than alone. On the other hand, effects like reduced neophobia, social facilitation, or more complex forms of SL can benefit individuals in groups over solitary individuals (43). If the positive and negative effects of the social environment cancel each other out, individuals in both conditions will be about equally successful (for humans, see the review in 41).

In this study, we used this virtual vs. real group approach to examine group problem-solving in cooperatively breeding common marmosets (*Callithrix jacchus*). Although social tolerance can vary with sex and breeding status as well as food distribution (44, 45), marmoset monkeys show high social tolerance, as do other cooperatively breeding primates (46–50). Like humans, they regularly engage in cooperative behaviors during their everyday life, like sharing valuable food items or infant care (51, 52) and engage in highly coordinated activities like vocal turn taking (53, 54), and even co-represent each other's actions in a joint action task (55). Marmosets are very proficient social learners (48, 53, 56, 57). Passive diffusion of information in marmosets is mediated via simple SL mechanisms like social facilitation and enhancement (58), but also more complex mechanisms like imitation (59). Yet, even simple forms of SL in marmosets are guided by intention attribution in that marmosets only learn from individuals that they perceive as goal directed agents (58). Some evidence suggests that they also engage in more active forms of information transmission (53, 60). This transition from passive to active information exchange is arguably driven by motivational factors linked to cooperative breeding, predisposing them to share information more readily than independently breeding species (61, 62).

**Fig. 1.**
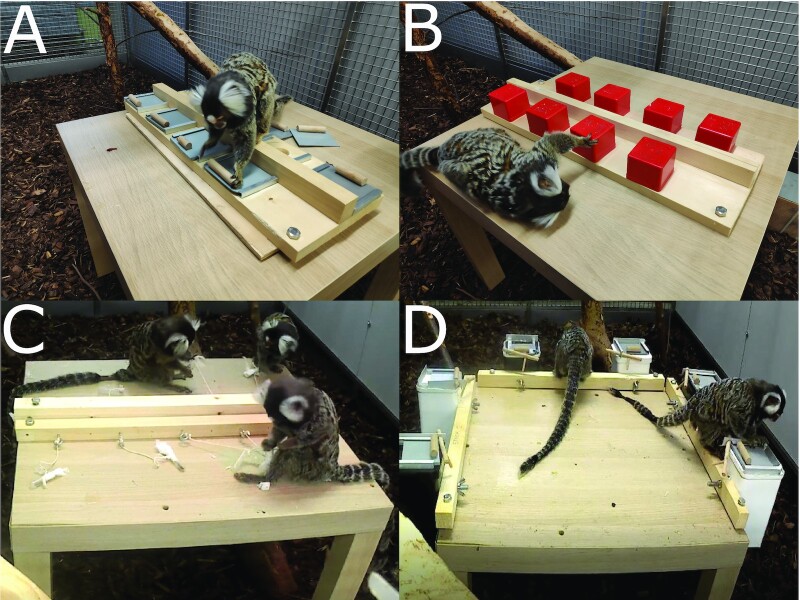
The four experimental tasks. (A) subject needs to pull a slider to reveal the food item; (B) subject needs to lift a red box and hold it until food item is taken; (C) subject needs to pull a cotton plug out of an Eppendorf tube and turn the tube upside down to retrieve the food item; and (D) subject needs to remove a wooden stick to unblock the slider that needs to be pulled next. In a last step, the subject needs to pull up a string to retrieve the food item attached at the end of the string.

We presented four novel tasks to both groups and single individuals (Fig. 1; Appendix Movie S1). We designed the tasks in a way that each subsequent one required more manipulation steps to obtain the reward: task difficulty thus increased from the first to the last task. The tasks were designed in a way that animals could combine potentially gathered information but also that each animal could solve a problem alone. Thus, our problems are best described as disjunctive tasks, where the problem is solved when a single solution is found (63). This approach was necessary to give each solitarily tested individual a chance to solve the problem alone. Hence, group success for both conditions is determined by the most skilled member, while it still allows us to observe individual success in each condition. Each task was presented (both to the groups and the individuals) for a maximum of 10 sessions of 5 minutes each, on separate days. For most groups, each individual was alternately tested in its own group and alone in an equal number of tasks (see Appendix Table S1 for details).

Our goal was to evaluate differences in problem-solving between individuals tested in the real groups and individuals tested in the virtual groups, and with what mechanism of the collective problem-solving continuum (Table 1) they would achieve that. Thus, we examined whether individuals in real groups were more likely to solve a novel problem or did so faster in the time allotted in the experiment than individuals tested in the virtual groups. We were also interested in whether groups were disproportionally more successful than individuals tested alone. For that we looked at whether real groups repeatedly solved more problems than virtual groups. Since common marmosets differ in personality traits that might also influence problem-solving ability, like for instance boldness (64, 65), we expected to find variation in individual innovative propensity and thus likelihood of success. As in humans, marmoset groups might contain members that are high performers by any standard (29, 66). Thus, we were particularly interested in whether groups would outperform even the best performing individuals tested alone.

Next, we investigated where on the collective problem-solving continuum common marmosets would most likely be situated (Table 1). Therefore, we quantified how potential candidate processed would facilitate problem-solving performance, namely (i) the social facilitation of neophobia, (ii) stimulus enhancement effects on exploration and more precisely (ii-a) how many interactions were needed until a task was solved; and (ii-b) for how long animals would explore the problem if they failed to solve the task, and (iii) whether a longer perseverance was socially induced by the success of a conspecific within the real group and elicited more goal directed exploration in yet unsuccessful individuals.

## Results

### Are individuals in real groups better problem solvers?

We presented four tasks with increasing difficulty to 25 common marmosets, in both real groups and as separate individuals (see the “Materials and methods” section). Overall, we recorded 5539 solving attempts (*n* = 3809 in real group conditions; *n *= 1730 in virtual group conditions). A total of 1225 of these attempts were successful and resulted in access to the food in a device (*n *= 740 in the real groups; *n *= 485 in the virtual groups).

We first examined the probability of individuals in real groups to solve a problem at least once compared to individuals in virtual groups. We combined the results over all tasks and ran a generalized linear mixed model with binomial error structure. We used condition (real vs. virtual group), sex, status (breeder vs. helper), and task as fixed factors and individual ID nested in family ID as random factor. We specified a priori contrasts for the factor task to perform polynomial trend analyses, given that we expected an increase in task difficulty from the first to the last task. Note that an animal could have solved one task but not another and will be treated as solver and nonsolver accordingly. The odds ratio for condition was significantly larger than one (odds ratio = 7.53, CI_95%_ = 2.17 to 26.16; *P* = 0.001; Appendix Fig. S1), indicating that individuals within the real groups were more than seven times more likely to solve a task than individuals within the virtual groups. No other predictors in our model achieved statistical significance, although breeders tended to show higher odds of success, and success tended to decrease with increasing task difficulty (Appendix Table S2).

**Fig. 2.**
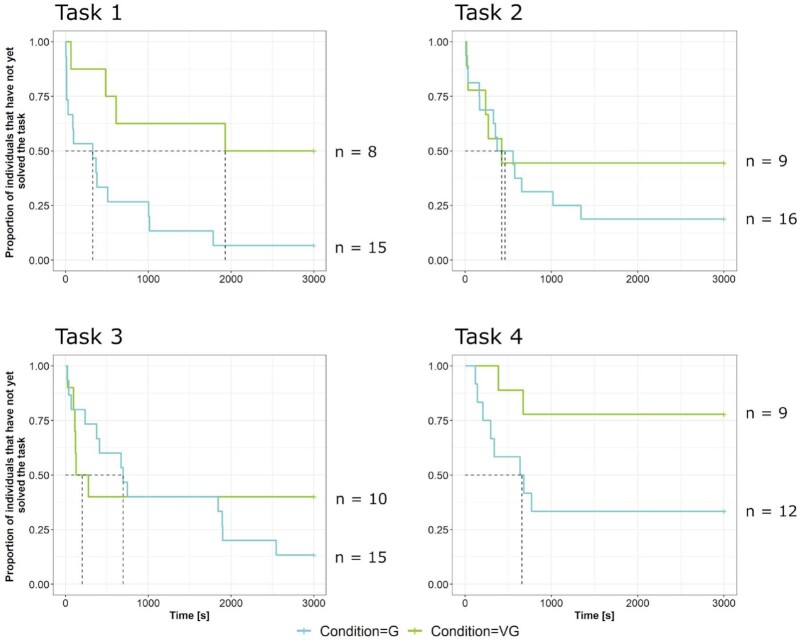
Survival plots showing the effect of the condition (G = real group, VG = virtual group on individual solving latency. Individuals within the real group found the solution to a given device earlier than animals in the virtual group. Moreover, the proportion of animals that had found the solution in the given time was always higher in the real group compared to the virtual group. Dashed lines indicate the median survival pointers (for details, see Table S4). The number of animals (*n*) per condition is indicated on the right side of each plot.

We next calculated a mixed effects Cox regression model for latencies until solution (Appendix Table S3). The strongest effect was observed between conditions, i.e. animals within a real group solved their first device far earlier than animals in the virtual group (proportional hazards ratio = 3.60, CI_95%_ = 1.77 to 7.30, *P* < 0.001; Fig. 2). Individuals within a real group were 3.6 times more likely to solve a problem than individuals within the virtual group at a given instant in time. In all tasks, individuals in the real group were on average faster than individuals in the virtual groups to find the solution, and in the first and the last task they were so significantly (proportional hazards ratio = 9.99, CI_95%_ = 1.34 to 74.63, *P* = 0.025; proportional hazards ratio = 12.17, CI_95%_ = 1.67 to 88.44, *P* = 0.01). In each condition, individuals took longer to solve later, more difficult tasks than the earlier ones (proportional hazards ratio = 0.41, CI_95%_ = 0.22 to 0.73, *P* < 0.01), and helpers were slower at solving their first device than breeders (proportional hazards ratio = 0.32, CI_95%_ = 0.14 to 0.72, *P* < 0.01).

We also compared the proportion of success of real and virtual groups measured as the number of extracted food items divided by the number of food items that could potentially be extracted maximally, for all group members and over all sessions and tasks. We analyzed how many of the tasks were successfully solved in each condition. For the real groups the proportion of success equaled the total number of extracted crickets. For the virtual groups, we summed the individual success (*s*) of each individual in the virtual group (*n*) and divided this value by the total number of devices (*d*) that could potentially have been solved by the respective individuals as in [1].

(1) \begin{equation*} \mathop \sum \limits_{i\ = \ 1}^n {s}_i*\ \frac{1}{{n*d}} = \rm {proportion\ of\ success\ of\ virtual\ groups}. \end{equation*}

**Fig. 3.**
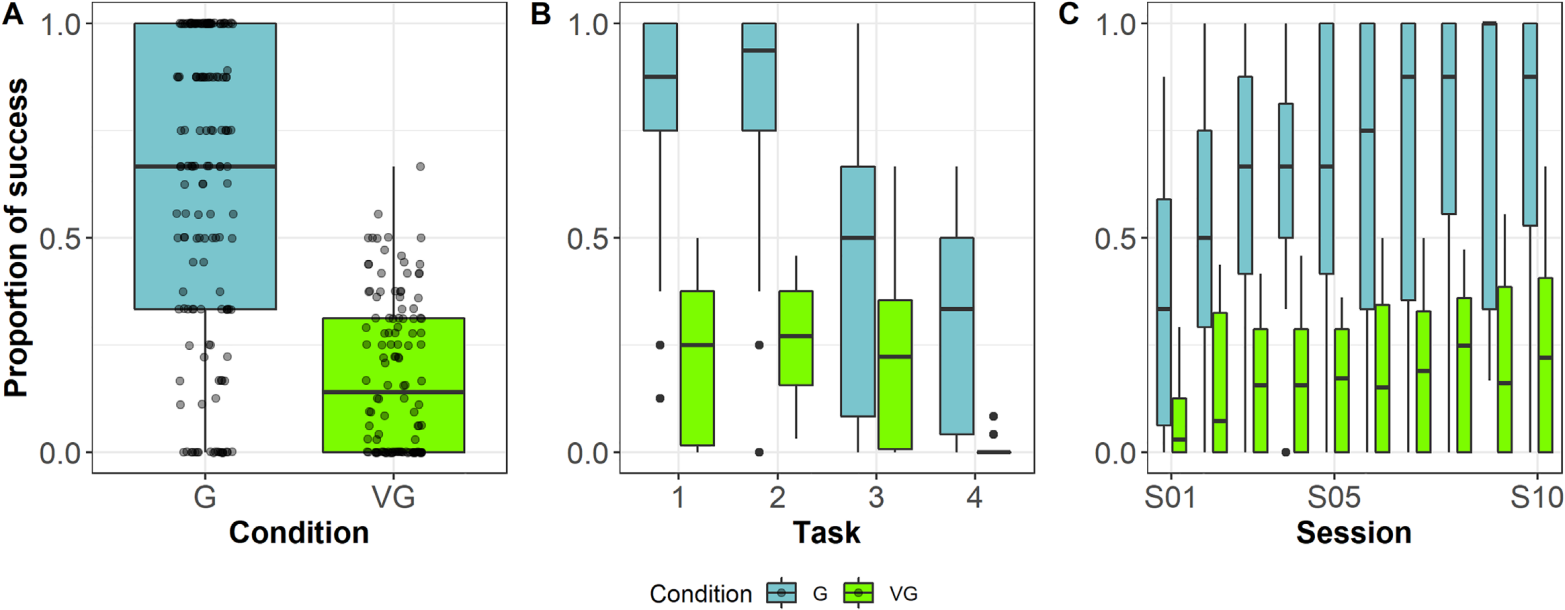
Comparison of proportion of success between real and virtual groups. (A) The percentage of solved devices from all possible devices over all sessions and all task between groups (G) and virtual groups (VG). (B) The amount of potentially solved devices decreased with increasing task complexity in both conditions. The strongest effect is notable in the most difficult task. (C) The proportion of potentially solved devices increased with increasing session number in both conditions. Note that due to their good performance in the first and second task, groups often performed at ceiling from session five onward. Black lines indicate medians; upper and lower edges of boxes indicate upper and lower quartiles; whiskers indicate the ranges for the bottom and top 25% of the data values, excluding outliers; transparent dots in (A) indicate raw values; and black dots in (B) and (C) indicate outliers.

We used a generalized linear mixed model with a binomial error structure and logit link function to explore the probability of success over all sessions (Appendix Table S5). Condition had a significant effect on overall success (Fig. 3A): individuals in real groups solved a larger proportion of devices than individuals in the virtual groups (odds ratio = 13.09, CI_95%_ = 10.13 to 16.91, *P* < 0.001). Although the proportion of success drops in both conditions with increasing task difficulty (odds ratio = 0.11, CI_95%_ = 0.34 to 0.52, *P* < 0.001; Fig. 3B), individuals in the group condition significantly outperformed individuals in virtual groups in three of the four tasks (only the third task showed no difference). The biggest effect was observed in the fourth, most difficult task, where real groups massively outperformed virtual groups (Fig. 3B). Only two animals within the virtual groups had solved this problem and each of them just once compared to eight individuals within the real groups who solved the problem 51 times. The odds of success increased with session number, suggestive of learning over repeated exposure (odds ratio = 3.86, CI_95%_ = 0.29 to 5.10, *P* < 0.001, Fig. 3C). Moreover, a post-hoc analysis of the interaction between session and condition revealed that the learning curve was steeper in the real groups compared to the virtual groups (odds ratio = 1.86, CI_95%_ = 1.05 to 3.30, *P* < 0.03).

**Fig. 4.**
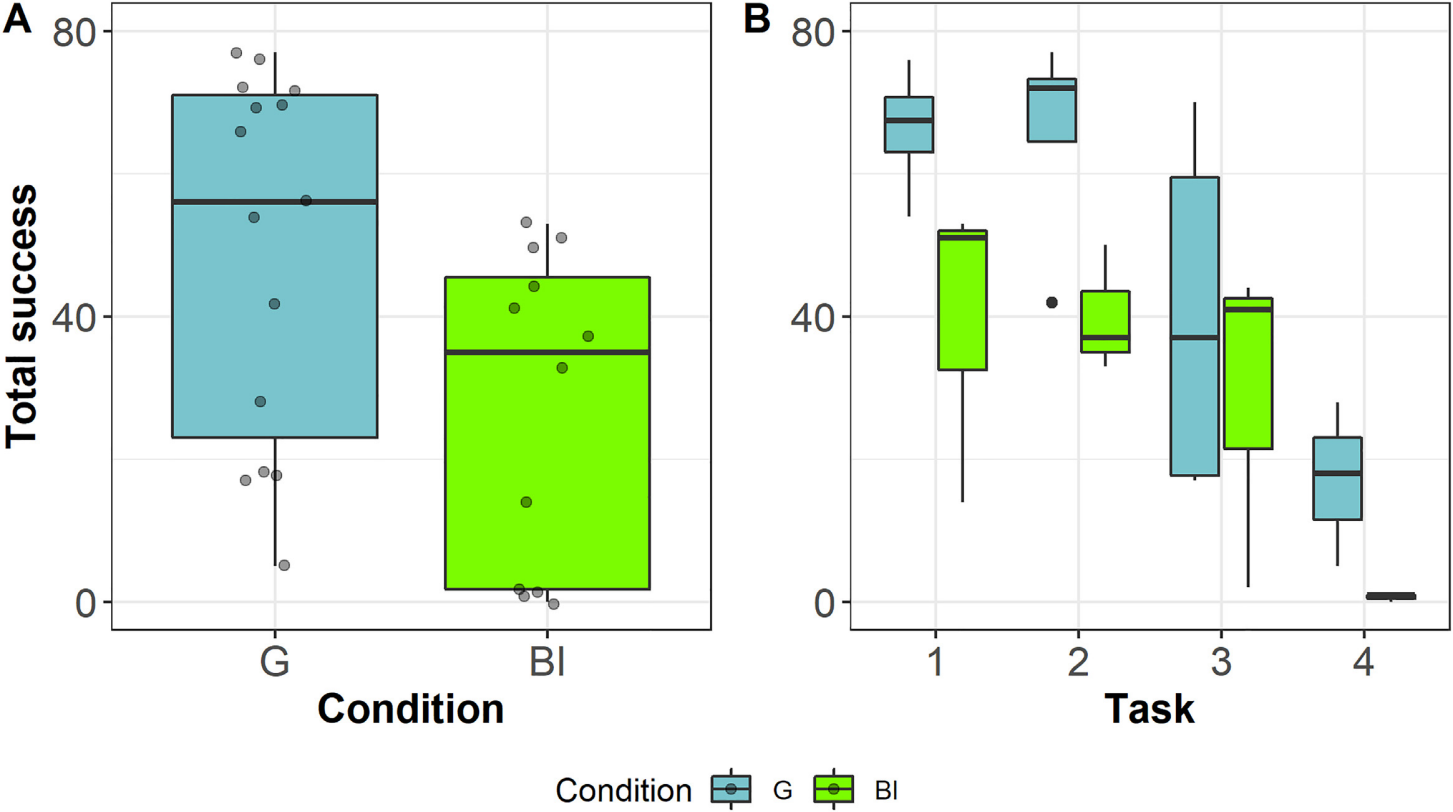
Comparison of success between real groups and best individuals of virtual groups. (A) Comparison of success measured as total number of extracted food items over all sessions between groups and the best individuals (BI) of the virtual groups. (B) Comparison of success between groups and best individuals split by task difficulty. Groups outperformed the best individuals of the virtual groups in three out of four tasks and outperformed the best individuals massively in the most difficult task. Black lines indicate medians; upper and lower edges of boxes indicate upper and lower quartiles; whiskers indicate the ranges for the bottom and top 25% of the data values, excluding outliers; transparent dots in (A) indicate raw values; and black dots in (B) indicate outliers.

As expected, individual differences were appreciable. Thus, we were interested in whether the superior performance of the group was an artifact driven by poorly performing individuals in the virtual groups. We therefore also compared the best performing individuals (BI) of each virtual group to the performance of the real groups. A mixed effects Cox regression model revealed that there was no difference between the latency of the real group to find the first solution and the latency of the best individual of each virtual group (odds ratio = 0.43, CI_95%_ = 0.84 to 6.57, *P* = 0.11). However, over all sessions, groups outperformed even these best solitary individuals by solving more devices (odds ratio = 6.54, CI_95%_ = 1.84 to 23.21, *P* < 0.01; Fig. 4A; note that several individuals have shown that the time available to single individuals was sufficient for them to solve all devices and consume all rewards). Again, the effect increased with task difficulty (odds ratio = 0.08, CI_95%_ = 0.06 to 0.11, *P* < 0.001; Fig. 4B).

### What makes real groups better problem solvers?

Individuals in real groups clearly outperformed individuals in virtual groups regarding proportion of success, and individuals in the real groups were more likely to find the solution and did so faster. We therefore wanted to pinpoint the mechanisms that caused these advantages of solving a problem within the group in common marmosets. The potential mechanisms we scrutinized were neophobia, exploration tendency, and perseverance, as well as the effect of observing a group member accessing the food in a device on the motivation of nonsolvers to interact with the device (socially induced perseverance) and on the goal-directedness of those interactions. First, we analyzed whether these factors differed between the two conditions and then tested whether they would predict problem-solving success of the real group.

### Animals in the real group showed reduced neophobia

**Fig. 5.**
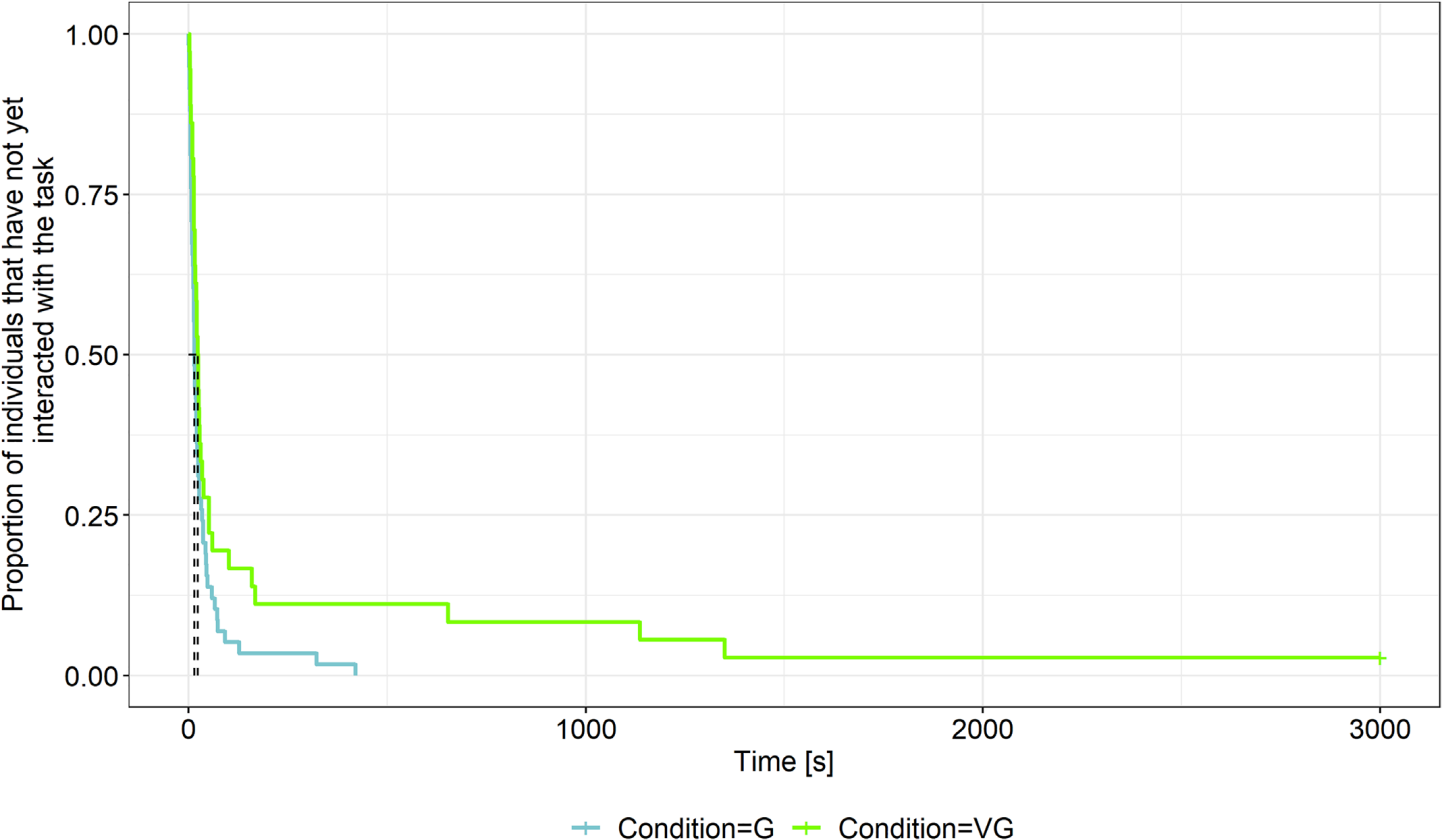
Survival plot showing the effect of a social environment on individual neophobia. Animals within a real group (blue) had shorter latencies to interact with a given device than animals in a virtual group (green). Dashed lines indicate the median survival pointers showing that the median latency to approach a task for the first time were very similar, 15.2 s for individuals in real groups and 24.3 s for individuals in virtual groups.

Except for one individual in the virtual group condition during the third task, all individuals interacted at least once with all tasks. We fitted a mixed effects Cox regression model to explore differences in neophobia between real and virtual groups (Appendix Table S6). Animals tested in a real group approached a device significantly faster than their counterparts tested in the virtual group (proportional hazards ratio = 2.00, CI_95%_ = 1.18 to 3.41, *P* = 0.01; Fig. 5). Hence, an individual in a real group was two times more likely to approach a device at any given time compared to an individual in a virtual group. The effect of neophobia was strongest in the first task where the setup was new to all animals (Appendix Fig. S2). Here individuals within the group were clearly faster in approaching the novel object compared to animals in the virtual groups (proportional hazards ratio = 3.79, CI_95%_ = 1.24 to 11.63, *P* = 0.02). The probability that an animal in the group had approached the first task at a given instant in time was 3.8 times higher than for an animal within the virtual group. However, the effect decreased from task to task and had worn off by the last task. Intriguingly, when we integrated the latency of first approach as fixed factor in our model to predict whether an animal had solved the task at all, it was not significant and the odds of solving were almost identical (odds ratio = 0.99, CI_95%_ = 0.98 to 1.00, *P* = 0.11). Neophobia thus differs between individuals tested alone or in a group, but it does not contribute significantly to the increased problem-solving success of the real groups compared to the virtual groups.

### Real groups and virtual groups did not differ in the amount of exploration needed to solve a problem

**Fig. 6.**
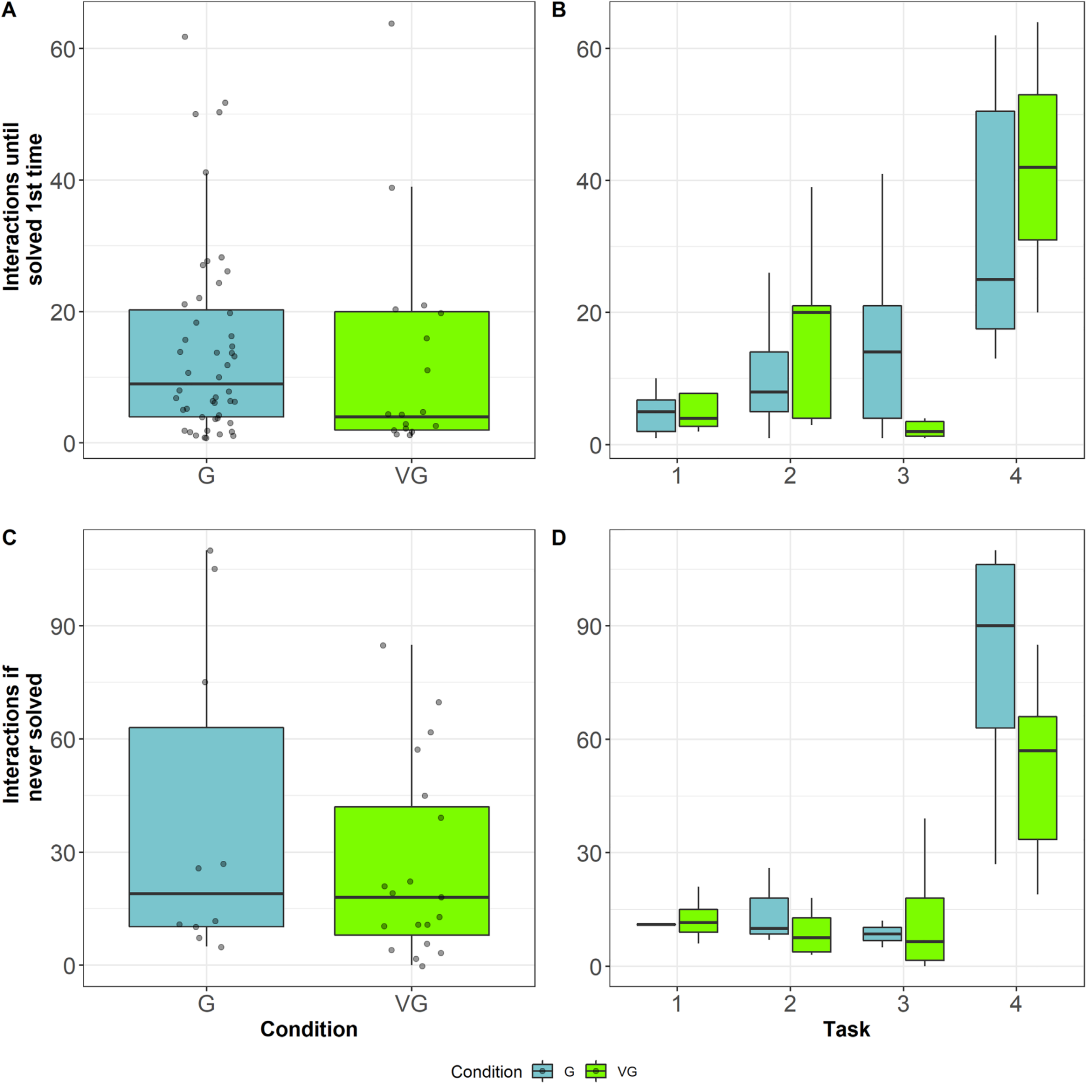
Levels of exploration in real and virtual groups. (A) The comparison between groups (blue) and virtual groups (green) revealed no difference in exploration attempts needed until a task was solved for the first time. (B) The necessity to explore possible solutions increased with increasing task difficulty and peaked with the most difficult task. (C) In real groups, individuals that never solved the presented task showed a higher perseverance (number of interactions) than individuals in virtual groups.(D) The perseverance of individuals was highest for the most complex task and individuals within a group explored this task 50% more often than individuals within virtual groups. Black lines indicate medians; upper and lower edges of boxes indicate upper and lower quartiles; whiskers indicate the ranges for the bottom and top 25% of the data values, excluding outliers; and transparent dots in (A) and (C) indicate raw values.

We calculated a generalized linear mixed model with a Poisson distribution for the number of interactions that an individual needed to solve a device for the first time (Appendix Table S7). Condition did not significantly contribute to the exploration rate and was close to identical between conditions. Although fewer individuals within the virtual groups solved their task, for those who did, the exploration rate before solving it was similar to the individuals within the real group (odds ratio = 0.96, CI_95%_ = 0.78 to 1.19, *P* = 0.73; Fig. 6A). Only task difficulty predicted the number of interactions an animal needed to find the solution. The more difficult the task the more interactions were needed to solve it (odds ratio = 3.07, CI_95%_ = 2.60 to 3.61, *P* < 0.01; Fig. 6B).

### Individuals within real groups showed a higher perseverance and were particularly motivated when a conspecific solved a task

We calculated a generalized linear mixed model with Poisson distribution for the number of interactions an individual performed if it never solved a problem (Appendix Table S8). Note that an individual's inclusion in the analysis of exploration rate or perseverance is task-specific and depends on whether it solved the task. For instance, an animal that had only solved the first two tasks but not the latter would be included in the exploration analysis for task number one and two and in the perseverance analysis for task number three and four. Perseverance of an individual was predicted by condition: animals within a real group showed a higher motivation than animals within a virtual group to interact with a device even if they had not solved it (odds ratio = 1.74, CI_95%_ = 1.24 to 2.43, *P* < 0.001; Fig. 6C, and D).

**Fig. 7.**
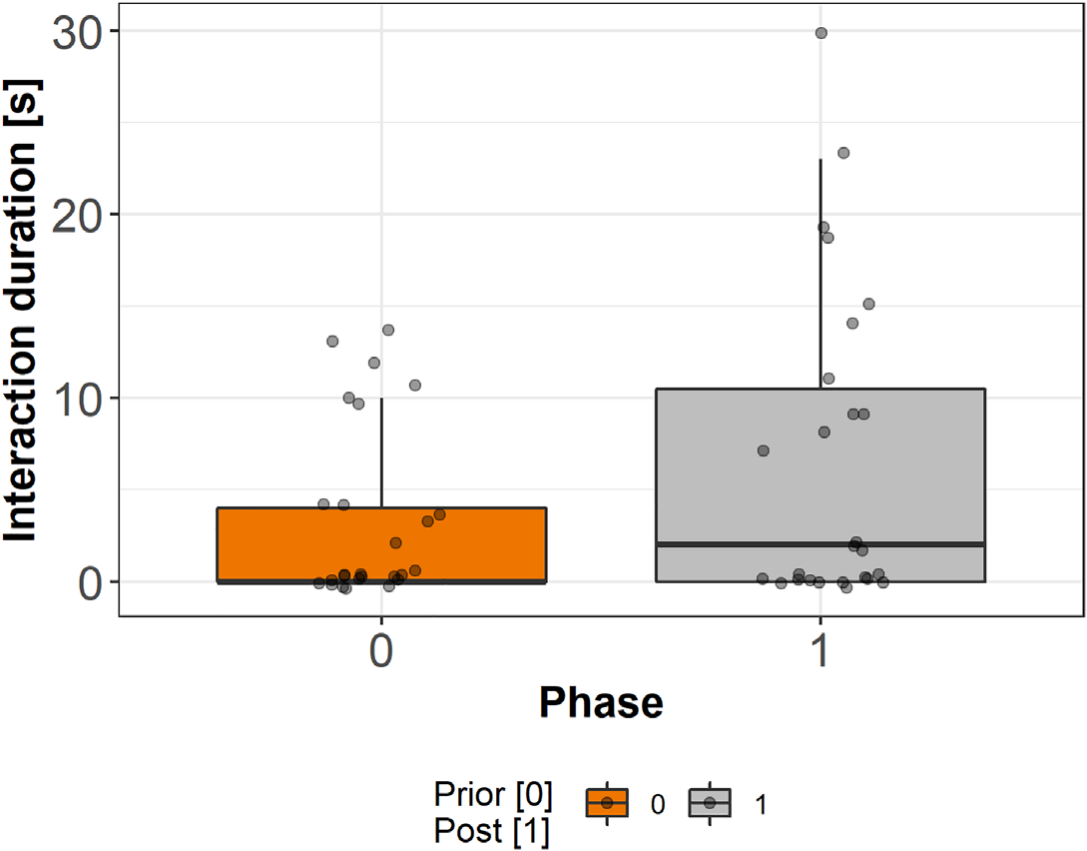
Exploration duration during the 30 seconds prior and post having observed a conspecific solving a task for the first time within the group condition. Black lines indicate medians; upper and lower edges of boxes indicate upper and lower quartiles; whiskers indicate the ranges for the bottom and top 25% of the data values, excluding outliers; and black dots indicate raw values.

We next asked whether the success of conspecifics within the real group had an immediate effect on the exploration rate of as yet unsuccessful individuals, and so increased their perseverance. Specifically, we analyzed their behavior 30 seconds before and after the first time a device was solved within a group (we had to exclude task 1 from this analysis because groups solved it too fast to analyze prior and post solving phases). We calculated a generalized linear mixed model (GLMM) with binomial error structure and logit link function. We used the proportion of interaction time during those phases as response variable and used only the phases (prior and post solving) as fixed effect and used identity nested within the family unit as random effect (Appendix Table S9). The phase did predict the interaction duration: animals that had not yet successfully solved the task increased their exploration tendency immediately after observing a conspecific solving a device (odds ratio = 2.77, CI_95%_ = 2.01 to 3.80 *P* = 0.001; Fig. 7).

## Discussion

The presence of group members can influence an individual's problem-solving ability in two ways (Table 1). It can negatively affect its propensity to innovate due to competition, scrounging and tactical deception or it can enhance its ability to solve a novel problem due to access to social information and a generally safer environment. A combination of both negative and positive effects could potentially cancel each other out, keeping the problem-solving ability approximately constant in social and asocial environments (67). Thus, group living species can be classified along the collective problem-solving continuum, depending on their intragroup competition and the prevailing mechanism of SL.

Our results show that the presence of conspecifics facilitates problem-solving in common marmosets (*C. jacchus*). Individuals in a real group were not only faster in solving a novel problem but also more likely to solve it at all. Thus, although we cannot exclude negative effects of intraspecific competition on certain individuals and their problem-solving propensity, the benefit of the social environment still increases the maximum achievable task complexity that an individual marmoset is able to solve above their baseline probability. Animals within a real group successfully accessed more devices and thus received more rewards than a comparable number of solitary individuals within their respective virtual group. Individuals that would otherwise struggle to cope with cognitively challenging novel problems benefited from the social information provided by more capable individuals. In fact, even the best individuals benefited from the presence of their conspecifics during complex problems. Hence, groups outperformed even the best individuals of each virtual group, a characteristic previously only known in humans (3–5), as the solitary individuals were mostly incapable of solving the most complex task alone. Although we cannot completely rule out the possibility that individuals tested alone were saturated with less than the maximum amount of possible food items, we think that this explanation is highly unlikely. We used crickets as food rewards, which are a highly preferred protein source for marmosets and several individuals in both conditions have shown that it is possible for a single monkey to devour all crickets in less than five minutes.

The major benefits of solving a problem in a group are most likely linked to a richer environment in terms of social information (43, 68). In this case we examined neophobia, which in animals does not always show a clear link to social environment or problem-solving (43, 31–33, 36, 69–72). Our results are in line with the general idea that the proximity of conspecifics reduces individual neophobia as a result of social facilitation (43, 72). However, although the animals' latency until solution was influenced by their latency until approach, ultimately neophobia did not predict whether an animal would solve the problem. In this study the time provided was long enough for even the more neophobic animals to still have enough time to find the solution. Among callitrichids, marmosets are considered neophilic (73) and do not necessarily rely on the presence of conspecifics to interact with novel objects. Thus, a reduction of neophobia may be beneficial for easy problems whose availability is limited in time but can be ignored otherwise.

We then examined factors related to exploration. Successful animals in both real and virtual groups did not differ in the amount of exploration they performed until they solved a problem. A likely explanation for this result is the influence of stimulus enhancement in combination with high degrees of social tolerance for individuals within real groups. Animals in real groups were able to simultaneously interact with the devices (see Appendix Movie S1). Thus, rather than waiting for other group members to interact with the device and learning about the properties of the problem by observation, each animal immediately began to explore it on its own. This is supported by the rather short latency of first approach within the group. Before individuals within the real groups could benefit from a successful group member, they often had already explored the device several times on their own. Hence, until the solution was found in the real group, each individual tried to find the solution via trial-and-error learning as it was the case in the virtual groups.

In the next step, we explored differences in perseverance between the two conditions. Among animals who were never successful in a given task, those within a real group had made more attempts during the available time than unsuccessful animals in the virtual group. Thus, animals within a group showed higher perseverance than individuals tested alone. This perseverance cannot simply be attributed to social facilitation or stimulus enhancement but was specifically induced by the success of other individuals. Individuals in the real group increased their interaction rate with the task immediately (within 30 seconds) after the first group member had solved the problem. Both social facilitation and stimulus enhancement would have a more general effect, and would not raise the individual exploration rate specifically after other individuals had solved the problem (74). Marmosets are known to engage in action observation and cognitively more demanding forms of SL like imitation (59, 75). In particular, in this species, even simple forms of SL such as social facilitation and stimulus enhancement rely on intention attribution (58), which may also have been responsible for this pattern of socially induced perseverance.

Studies of group problem-solving in the past have either focused on humans or used group size comparisons. Here we established a new way to examine these questions in nonhuman animals. The comparison of individuals within their native group with individuals tested solitarily allowed us to disentangle the baseline probability of an individual to solve a problem compared to the probability of solving a problem within a social environment. Although previous studies have tested animals in social or asocial environments during problem-solving, to our knowledge, none of them have pooled the performance of the solitary individuals to compare an equal number of animals. By pooling the solitarily tested individuals, we can test for effects that go beyond the sampling bias that is responsible for the pool of competence effect when comparing groups of different sizes (39). Low-level passive diffusion of social information combined with low levels of intragroup competition appears already sufficient for individuals in a group to gain an advantage over solitary animals. More sophisticated SL mechanisms like imitation, information donation, and ultimately the division of labor may lead to what we define as “true collective problem-solving” (Table 1).

Our experiments were not designed to distinguish whether marmosets that subsequently solved the task did so by imitating the movements of the successful ones. Neither could we examine whether there was active information exchange between group members. Although we could not properly test whether animals specialized on specific parts of the problem and engaged in something like “division of labor,” during interactions with the most complex task, we occasionally observed that some animals were better at removing the barricade and pulling the slider, whereas others were more apt at pulling up the strings. To further investigate collective problem-solving in marmosets, future studies should consider to design conjunctive tasks in which a task can only be solved when all members of the group have completed their portion of the problem. Given that marmosets are capable of true imitation (59) and appear to have some motivational predisposition for information donation (61), we consider it worthwhile for future studies to integrate tasks where one can distinguish between those more sophisticated mechanisms of SL and lower-level ones. As wild marmosets also have shown to learn from video material (56), future studies could also test real and virtual groups using recorded problem solvers and thus test the importance of social facilitation. Lastly, our study investigated captive common marmosets in a less threatening environment, where animals potentially can spend more time exploring novel objects than in the wild. Although studies on captive callitrichids have shown that they still engage in behaviors from the wild like antipredatory behaviors (76, 77), we cannot rule out the possibility that wild marmosets in groups would explore devices differently, for instance show higher neophobia or be more competitive over valuable resources or spend less time in total on complex problems.

The available data suggest that other nonhuman primates fall below marmosets in the collective problem-solving continuum (78). A recent study in chimpanzees has shown that although the innovative behavioral repertoire in groups was higher than in solitary individuals, the overall complexity of these innovations was not. The chimpanzees in both conditions repeatedly failed to solve the most complex task, although it was the most rewarding one (79). In the despotic rhesus macaques subordinate individuals actually hide their knowledge in the presence of more dominant animals (80), which suggests that groups would do even worse than individuals (see Table 1). Applying this approach to other species will allow us to obtain a more comprehensive picture of the evolution of collective problem-solving and its underlying mechanisms. Filling in these gaps may help us to understand why “true collective problem-solving” in humans is unique and how it integrates into the evolution of cumulative culture (2).

In the current study, marmosets not only solved the most complex task within the group, while solutions were virtually absent among the animals tested alone, but also allowed all others to participate, independent of dominance. One striking difference between callitrichids and other nonhuman primates is their rearing system. Like humans, callitrichid monkeys are cooperative breeders with high levels of proactive prosocial behavior and social tolerance (45–47, 49, 81). The high level of social tolerance protects the innovator to some degree from exploitation by conspecifics (Fig. S3). In contrast, in more competitive species individuals who spend a lot of time and energy to arrive at an innovative behavior are at risk of being displaced before they can reap the rewards (21, 50, 82). The marmoset monkeys in our study not only worked in close spatial proximity on multiple devices simultaneously but several monkeys regularly worked simultaneously on the same device (Appendix Movie S1). In fact, we almost certainly even underestimated the effect of social tolerance in our captive study as it was recently shown that due to a higher level of interdependence their wild counterparts exhibit higher levels of spatial- and feeding tolerance (46), an effect that may be limited to cooperative breeders.

In conclusion, our study shows that cooperatively breeding common marmosets share with humans the ability to solve even complex problems better in a group than alone. Marmosets, and arguably all callitrichids, thus offer a plausible and highly informative model for the evolution of human problem-solving abilities and the evolution of human culture. Human culture exceeds animal culture not only in its diversity but also in its unique complexity (1, 79, 83–86). Early hominins with their already high cognitive capacity may have benefited from this cooperative nature and collectively invented complex behaviors. Cooperative breeding in marmosets is linked to several characteristics that enhance cooperation in other domains as well (49, 52, 55, 62). Cumulative cultural evolution requires another key component, which is high-fidelity transmission of information between generations (84, 85). In great apes, in contrast, both the ability to solve problems collectively and high-fidelity information transmission mechanisms like teaching are absent (87–89) or rare (90, 91). And although the exact mechanisms are still under debate, marmosets show signs of high-fidelity information transmission (56, 59, 92). Hence, studies investigating potential candidates for group problem-solving should examine whether, and if so how, collectively obtained skills and information are transmitted within and across generations.

## Materials and methods

### Subjects

We tested seven groups of two to four individuals (*n* = 25 adults; see Appendix Table S10 for a list of animals in the experiment) of common marmosets (*C. jacchus*). All animals were born in captivity and housed in heated indoor enclosures with ad libitum access to outdoor enclosures if weather conditions allowed. Indoor and outdoor enclosures are highly enriched with wooden branches, ropes, diverse climbing structures, and soil with natural vegetation (outdoor) and wood chips (indoor). Animals are fed twice a day (vitamin-enriched porridge in the morning and fruits and vegetables at midday) and have access to water ad libitum. In addition, animals were given an afternoon snack composed of either animal protein (e.g. fish, boiled egg) or gum arabic. Animals were tested either in the morning between the feedings or in the afternoon before the snack feeding. The research was approved by the Kantonales Veterinäramt Zürich (ZH232/19).

### Apparatuses

We tested all marmosets with four distinct problem-solving tasks that varied in the number of necessary manipulation steps and therefore difficulty (Fig. 1A to D; Appendix Movie S1). For each task, multiple replicate devices were presented, so that multiple individuals could work simultaneously. We offered eight replicas for the first two tasks, nine for the third and six for the last task. The number of replicas depended on the size of the apparatus, solving time per task and consumption time for a cricket. Note that crickets during the third task were devoured faster as we removed the legs so that they would fit into the Eppendorf tubes. The goal of each task was to extract a cricket (a highly favored food item). In the first task the animals must grab a slider and pull it, so that a food reward is revealed (Fig. 1A). All following tasks increased not only in the number of steps required but also included bimanual actions, which are generally associated with a higher complexity level (93). In the second task, the animals must lift a box to get the reward from below. The boxes will automatically drop down again if not constantly held up (Fig. 1B). Thus, the animal must use both hands, one to hold open the box, the other to retrieve the reward. In the third task, the animals must unplug a piece of cotton out of an Eppendorf tube to get access to a food reward. Afterward, they must retrieve the reward out of the tube, which requires turning the tube upside down and thus fine motor skills (Fig. 1C). Task four requires three separate steps to reach the solution (Fig. 1D). In this task, the animals must loosen a barrier (step 1) that blocks a slider, which must be pulled (step 2) to give access to an opening of the box. Finally, the animal must pull up a 15 cm string that is hanging inside the box to get access to the food reward (step 3). We placed a table of 45 cm height and 55 cm times 55 cm platform in each home enclosure (indoor) during the time of the study. The apparatuses were attached to the table before and removed right after each test session. Apparatuses were cleaned in between sessions.

### Procedure

Tests were conducted in the home enclosures of the respective group. Home enclosures were 2.5 m × 1.8 m × 3.5 m in size and enriched with natural climbing structures. Animals were tested either in groups or individually. If animals were tested individually, we separated a single individual in the home enclosure while the rest of the group was either in the outdoor enclosure or in an adjacent indoor enclosure. Whether a family was tested individually or as a group was pseudo-randomized for each task. For husbandry reasons, we could not randomize two groups, which always had to be tested as group and two individuals, which always needed to be tested solitarily (see Appendix Table S1 for details). We recorded a total of 510 sessions. A session started as soon as the experimenter put the apparatus on the table and no longer touched the device, and lasted for five minutes. We conducted two sessions per day and a total of 10 sessions per task in either group or individual condition. The order of the tasks remained constant for each group/individual. Note that if an animal or a group had not solved a task over the 10 sessions, we showed the group/individual the solution to ensure that for the following tasks each animal had seen the association between the devices and food. Thus, we put each animal in the same information state. Sessions were recorded with two cameras, one inside the enclosure (Somikon Action-Cam DV-3217) with focus on the table and one outside to record the whole enclosure (Sony Handycam FDR-AX43 Camcorder).

### Data scoring

All sessions were video recorded and analyzed with Mangold INTERACT software (Mangold International, 2020). All individuals were individually recognizable due to small tail shavings. We coded for each individual and session: latency, duration and number of interactions with the device, latency until the first problem was solved, and number of problems successfully solved. Hence, we knew for each real group when a device was approached and solved for the first time and how many interactions were necessary to do so. To get the same output for the virtual groups, we pooled the approaching latency, solving latency and interactions necessary for solving a task of each solitary individual of a family unit and treated them as a virtual group. Thus, for instance, the solving latency of the fastest animal of a family unit was equal to the solving latency of the respective virtual or real group. For the interobserver reliabilities, two independent observers coded 10% of all videos. Both observers were trained to the use the ethogram used by the main coder (first author) but blinded to the overall study goal. We used a pairwise comparison between the main coder and interobserver 1 (IO1) and the main coder (MC) and interobserver 2 (IO2). MC and IO1 reached an overall intraclass correlation coefficient (ICC3) of 0.78 with a CI_95%_ = 0.73 to 0.82 and MC and IO2 reached an overall ICC3 of 0.97 CI_95%_ = 0.95 to 0.98.

### Statistical analysis

All analyses were carried out using RStudio (94) with packages “survival” (95), “coxme” (96), and “lme4” (97). For all models we calculated first a biologically inspired full model and recalculated an optimized model excluding nonsignificant factors one by one for each. As trends and significances were not influenced by optimizing the model, we report results for our biologically inspired full models. Figures were drawn using either “survminer” (98) for survival plots or “ggplot2” (99) for all other plots. We calculated the IOR using the package “psych” (100) and reported the ICC3 two way mixed effect model for fixed raters.

To examine the probability of an individual solving a problem in a real group compared to solving it in a virtual group, we fitted a generalized linear mixed model with a binomial error structure. We calculated a mixed effects Cox regression model to explore differences in solving latency between animals in real groups and animals in the virtual group. Latency was measured as the time an individual needed to solve a device from the beginning of the first session. If an individual did not solve a problem within the first session, we assigned the five minutes plus the time the animal needed in the next session to solve a device, up to a maximum time of 3000 seconds (10 sessions). For neophobia, we used the latency of when an animal approached the device for the first time or we assigned the maximum time of 3000 seconds if an animal never approached a device. We used latency as response and condition, sex, status, and task as fixed factors and included the individual ID nested in family ID as random factor for both analyses. We used this structure of fixed and random effects for all our models unless specified differently. We specified a priori contrasts for the factor task to perform polynomial trend analyses, given that we expected an increase in task difficulty from the first to the last task. To analyze the overall success of groups and virtual groups, we combined the number of all devices successfully solved within a group or a virtual group as in [1] and calculated a GLMM with task and session as fixed factors and included family as random factor. We used the same approach as for the latency of first solved device to examine the latency of first approach between conditions to determine individual neophobia.

To analyze the difference in the exploration rate needed until a device was successfully accessed and perseverance, we counted the number of interactions with a device. Interactions with parts of already solved devices were not considered. We used the number of interactions until a device was solved as measurement for exploration rate needed and the number of interactions an individual had if the task was never solved as measurement for perseverance. We calculated GLMMs using the same fixed and random factors as for the mixed effects Cox regression model and used a Poisson distribution error structure. To further look at how much more likely an individual will interact with a problem after it observed a conspecific solving it, we examined the 30 seconds prior and post the moment a device was solved for the first time. We calculated a binomial GLMM with log link function using the duration of interaction within the 30-second segment and prior and post solving period as response. We included the individual ID nested in family ID as random factor.

Lastly, we analyzed which factors contributed to the likelihood of an animal solving a device at least once. We used success as binomial variable and included all previous fixed and random factors into our model plus neophobia and exploration tendency (until solved or total if not solved) as additional fixed factors and calculated a binomial GLMM with log link function.

## Supplementary Material

pgac168_Supplemental_Files

## Data Availability

All data needed to evaluate the conclusions in the paper are present on OSF (https://doi.org/10.17605/OSF.IO/7D8YT)
