# Supplementary material for: Problem-solving in groups of common marmosets (Callithrix jacchus): more than the sum of its parts
Source: PNAS Nexus. 2022 Sep 14;1(4):pgac168. doi: 10.1093/pnasnexus/pgac168 (PMC9802434; doi:10.1093/pnasnexus/pgac168)
Supplement: pgac168_Supplemental_Files [file pgac168_supplemental_files.zip › 562649_1_final_file_9759862_rg9p9l (1).docx]

**
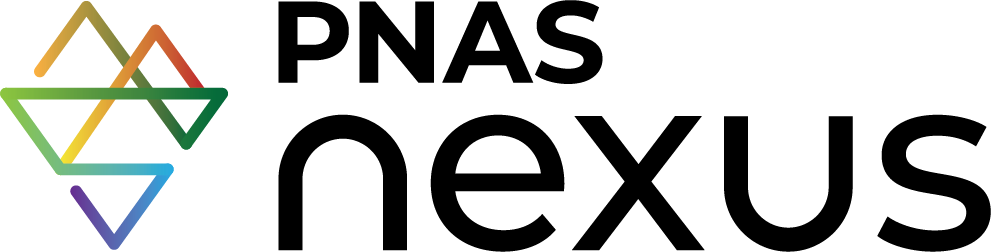
**

**Supplementary Information for**

Problem solving in groups of common marmosets (*Callithrix jacchus*): more than the sum of its parts

Sandro Sehner, Erik P. Willems, Lucio Vinicius, Andrea B. Migliano, Carel P. van Schaik, Judith M. Burkart

Sandro Sehner

Email: [sandro.sehner@uzh.ch](mailto:sandro.sehner@uzh.ch)

**This PDF file includes:**

Figures S1 to S3

Tables S1 to S10

Legends for Movie S1

**Other supplementary materials for this manuscript include the following:**

Movie S1


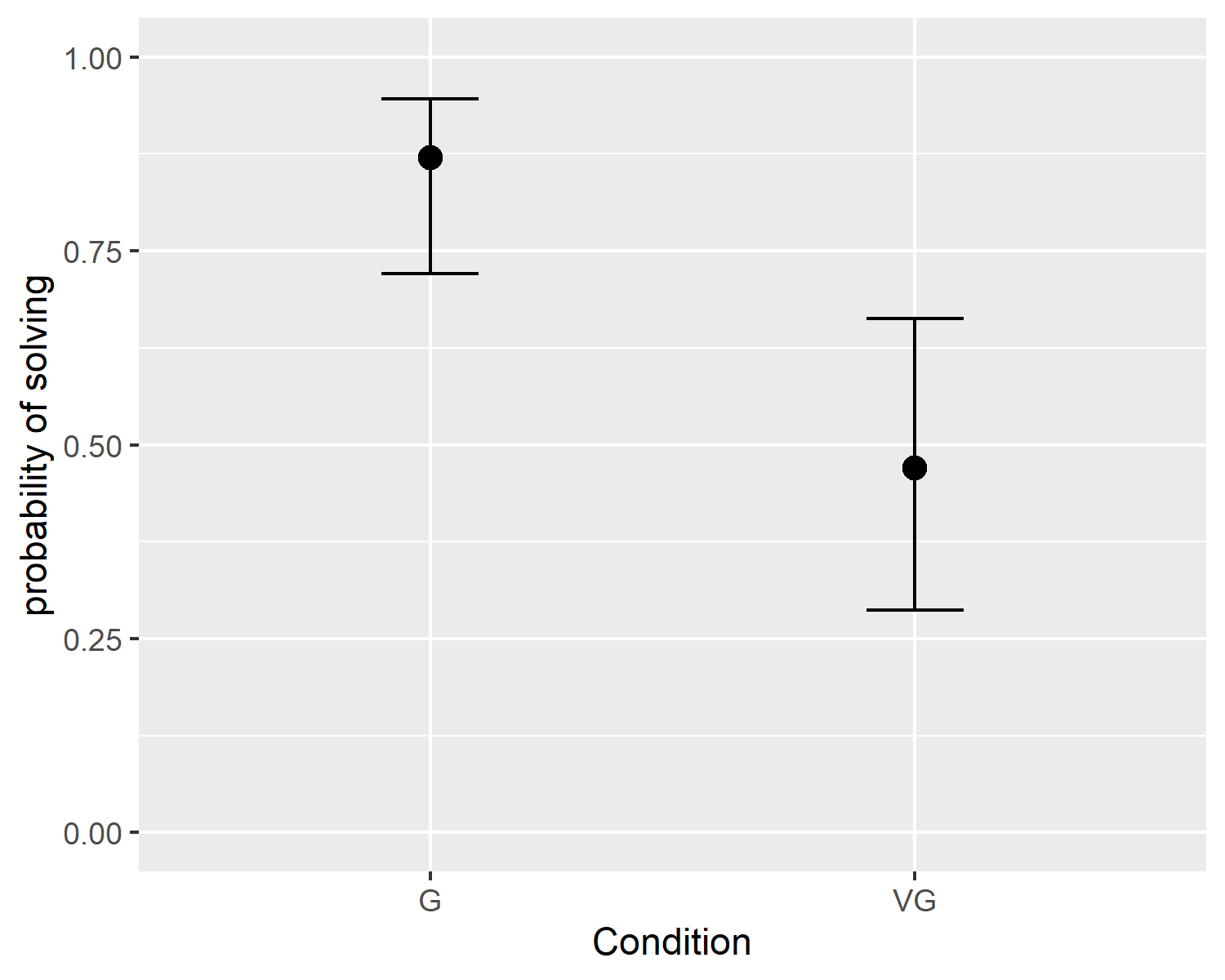


Fig. S1. Comparison of the probability of an individual solving a task within the real group [G] and the virtual group [VG] condition. Individuals within the real group have a higher probability to solve a given task when being in a real group compared to being alone. Indicated are the fitted model predictions and the confidence limits as error bars.


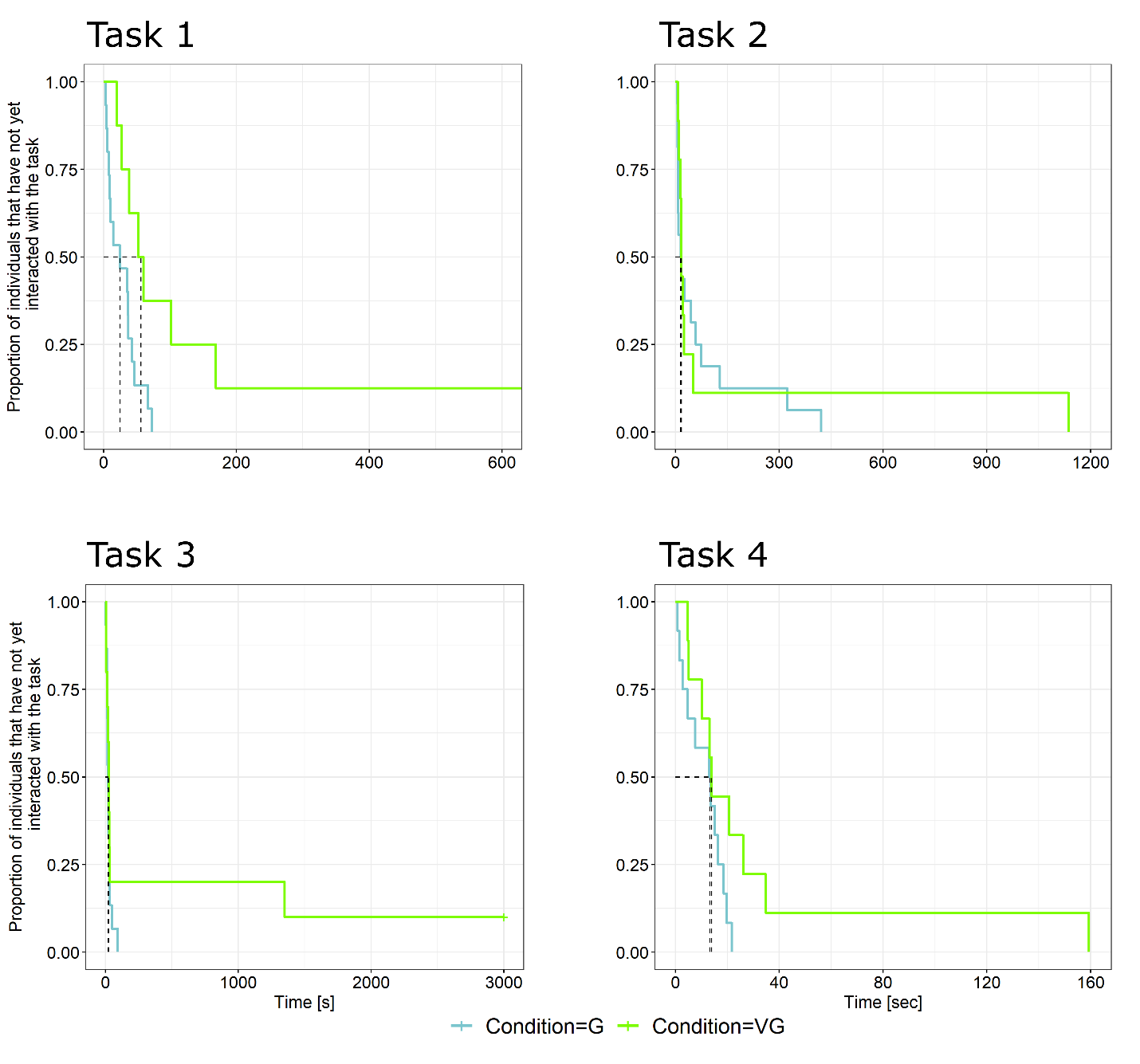
Fig. S2. Survival plots showing the effect of a social environment on individual neophobia separated by task. Animals within a real group (blue) interacted faster with the first task compared to animals in a virtual group (green). The effect decreased in the follow up tasks. Dashed lines indicate the median survival pointers. Median latencies to approach a device for real- and virtual groups were 24.6 s;56.1 s for task one, 15.5 s;16.8 s for task two, 20.2 s; 24.4 s for task three and 13.2 s;13.9 s for task four, respectively. Note that the scale for the x-axis is nonuniform.

Fig. S3. Comparing solved problems against rewards consumed per individual. Red line indicates a perfectly balanced relation between consumption and solving. Animals below have solved more problems than they consumed rewards, while animals above have consumed more than rewards than they solved problems. Note that there are no animals specialized on scrounging or providing but rather small variations.

**Table S1 Conditions of family units in which they were tested.** Animals in the virtual group condition were tested solitarily whereas animals in the group condition were tested within their family unit. Note that for husbandry reasons the last three groups were not pseudo-randomized but stayed in either group- or virtual group condition. The order of the task was fixed from task 1 to task 4 for each real- and virtual group, respectively.

| Group  (No of ind.) | Task 1  (slider) | Task 2  (box lifting) | | Task 3 (eppendorf tube) | Task 4 (complex box) |  |
| --- | --- | --- | --- | --- | --- | --- |
| Ninas (4) | Virtual  group | Group | Virtual  group | | Group |  |
| Minas (3) | Group | Virtual  group | Group | | Virtual  group | |
| Gabbanas (4) | Virtual  group | Group | Group | | Virtual  group | |
| Lancias (4) | Group | Virtual  group | Virtual  group | | Group | |
| Grappas (4) | Group | Group | Group | | NA | |
| Nalas (4) | Group | Group | Group | | Group | |
| Limas (2) | Virtual  group | Virtual group | Virtual group | | Virtual group | |

Table S2. Summary of the generalized mixed model for individual success measured as solved or not solved. Note that individuals that are solvers in one task can be non-solvers in another and vice versa.

| **parameter** | **odds ratio** | **95% CI** | **SE** | **z** | **p** |
| --- | --- | --- | --- | --- | --- |
| Intercept | 1.59 | [0.45, 5.60] | ±0.64 | 0.73 | 0.47 |
| Condition (G) | 7.53 | [2.17, 26.16] | ±0.63 | 3.18 | 0.001 |
| Sex (M) | 0.98 | [0.39, 3.48] | ±0.64 | -0.03 | 0.97 |
| Status (H) | 0.31 | [0.27, 3.48] | ±0.65 | -1.81 | 0.07 |
| Task | 0.37 | [0.12, 1.14] | ±0.56 | -1.74 | 0.08 |

**Table S3. Summary of the mixed effects Cox regression model for latencies until solution**. Bold values indicate significant predictors (p < 0.05).

| **parameter** | **proportional hazards ratio** | **95% CI** | **SE** | **z** | **p** |
| --- | --- | --- | --- | --- | --- |
| Condition (G) | 3.60 | [1.77, 7.30] | ±0.36 | 3.55 | **< 0.001** |
| Sex (M) | 0.75 | [0.34, 1.67] | ±0.40 | -0.69 | 0.49 |
| Status (H) | 0.32 | [0.14, 0.72] | ±0.42 | -2.72 | **< 0.01** |
| Task | 0.41 | [0.22, 0.73] | ±0.30 | -2.98 | **< 0.01** |

**Table S4. Summary of the median survival for solving latency.**

|  | **Median survival latency** | |
| --- | --- | --- |
| **Task** | **G** | **VG** |
| 1 | 329 s | 1929 s |
| 2 | 464 s | 425 s |
| 3 | 697 s | 202 s |
| 4 | 659 s | NA |

**Table S5. Summary of the generalized mixed model for proportion of solved problems for groups and virtual groups.** Bold values indicate significant predictors (p < 0.05).

| **parameter** | **odds ratio** | **95% CI** | **SE** | **z** | **p** |
| --- | --- | --- | --- | --- | --- |
| Intercept | 0.11 | [0.06, 0.22] | ±0.33 | 1.18 | 0.23 |
| Condition (G) | 13.09 | [10.13, 16.91] | ±0.13 | 19.4 | **< 0.001** |
| Session | 3.86 | [2.92, 5.10] | ±0.15 | 9.23 | **< 0.001** |
| Task | 0.11 | [0.34, 0.52] | ±0.14 | -15.77 | **< 0.001** |

**Table S6. Summary of mixed effects Cox regression model for latencies of first-time interacting with a device.** Bold values indicate significant predictors (p < 0.05).

| **parameter** | **proportional hazards ratio** | **95% CI** | **SE** | **z** | **p** |
| --- | --- | --- | --- | --- | --- |
| Condition (G) | 2.00 | [1.18, 3.41] | ±0.27 | 2.56 | **0.01** |
| Sex (M) | 0.74 | [0.42, 1.30] | ±0.28 | -1.05 | 0.29 |
| Status (H) | 0.56 | [0.31, 1.00] | ±0.3 | -1.94 | 0.052 |
| Task | 2.1 | [1.30, 3.38] | ±0.24 | 3.05 | **0.002** |

**Table S7. Summary of the generalized liner mixed model for number of interactions needed to solve a device for the first time.** Bold values indicate significant predictors (p < 0.05).

| **parameter** | **odds ratio** | **95% CI** | **SE** | **z** | **p** |
| --- | --- | --- | --- | --- | --- |
| Intercept | 10.25 | [6.6; 15.92] | ±0.22 | 10.6 | **< 0.001** |
| Condition (G) | 0.96 | [0.78, 1.19] | ±0.11 | -0.35 | 0.73 |
| Sex (M) | 1.24 | [0.86, 1.80] | ±0.19 | 1.16 | 0.25 |
| Status (H) | 1.17 | [0.81, 1.7] | ±0.19 | 0.85 | 0.39 |
| Task | 3.07 | [2.60, 3.61] | ±0.08 | 13.39 | **< 0.001** |

**Table S8. Summary of the generalized liner mixed model for number of interactions of animals that never solved a device.** Bold values indicate significant predictors (p < 0.05).

| **parameter** | **odds ratio** | **95% CI** | **SE** | **z** | **p** |
| --- | --- | --- | --- | --- | --- |
| Intercept | 11.68 | [5.76, 23.66] | ±0.38 | 7.88 | **<0.001** |
| Condition (G) | 1.74 | [1.24, 2.43] | ±0.17 | 3.22 | **0.001** |
| Sex (M) | 1.27 | [0.64, 2.53] | ±0.35 | 0.7 | 0.49 |
| Status (H) | 1.01 | [0.50, 2.03] | ±0.36 | 0.03 | 0.98 |
| Task | 2.81 | [2.22, 3.57] | ±0.12 | 8.60 | **<0.001** |

**Table S9. Summary of the generalized mixed model for interaction rate before and after first solving event within a group.** Bold values indicate significant predictors (p < 0.05).

| **parameter** | **odds ratio** | **95% CI** | **SE** | **z** | **p** |
| --- | --- | --- | --- | --- | --- |
| Intercept | 0.04 | [0.01, 0.1] | ±0.54 | -6.1 | **< 0.001** |
| Phase | 2.77 | [2.01, 3.8] | ±0.16 | 6.27 | **< 0.001** |
| Task | 2.07 | [1.17, 3.66] | ±0.29 | 2.51 | **0.01** |

**Table S10. List of individuals in the experiment.** Animals can be classified into four categories according to their sex and status

| **Group** | **Individual** | **Sex** | **Status** |
| --- | --- | --- | --- |
| Gabbanas | Gabbana | Female | Breeder |
|  | Lars | Male | Breeder |
|  | Garbo | Male | Helper |
|  | Gin | Female | Helper |
| Minas | John | Male | Breeder |
|  | Merkur | Male | Helper |
|  | Mojita | Female | Helper |
| Nalas | Nala | Female | Breeder |
|  | Tamino | Male | Breeder |
|  | Narnia | Female | Helper |
|  | Nirvana | Female | Helper |
| Grappas | Grappa | Female | Breeder |
|  | Craken | Male | Breeder |
|  | Guapa | Female | Helper |
|  | Ginger | Female | Helper |
| Ninas | Lex | Male | Breeder |
|  | Nougat | Female | Helper |
|  | Nux | Female | Helper |
|  | Nox | Male | Helper |
| Limas | Lima | Female | Breeder |
|  | Mars | Male | Breeder |
| Lancias | Lancia | Female | Breeder |
|  | Lexus | Male | Breeder |
|  | Lotus | Male | Helper |
|  | Lola | Female | Helper |

Movie S1.

Video sequences of common marmosets interacting and solving Tasks 1-4 within the real groups.
